# Molecular cloning and expression of a novel trehalose synthase gene from *Enterobacter hormaechei*

**DOI:** 10.1186/1475-2859-8-34

**Published:** 2009-06-12

**Authors:** Ming Yue, Xiu Li Wu, Wei Na Gong, Hong Biao Ding

**Affiliations:** 1 Feed Research Institute, Chinese Academy of Agricultural Sciences, Beijing 100081, PR China; 2 Institute of Plant Protection, Chinese Academy of Agricultural Sciences, Beijing 100094, PR China

## Abstract

**Background:**

Trehalose synthase (TreS) which converts maltose to trehalose is considered to be a potential biocatalyst for trehalose production. This enzymatic process has the advantage of simple reaction and employs an inexpensive substrate. Therefore, new TreS producing bacteria with suitable enzyme properties are expected to be isolated from extreme environment.

**Results:**

Six TreS producing strains were isolated from a specimen obtained from soil of the Tibetan Plateau using degenerate PCR. A novel *treS *gene from *Enterobacter hormaechei *was amplified using thermal asymmetric interlaced PCR. The gene contained a 1626 bp open reading frame encoding 541 amino acids. The gene was expressed in *Escherichia coli*, and the recombinant TreS was purified and characterized. The purified TreS had a molecular mass of 65 kDa and an activity of 18.5 U/mg. The optimum temperature and pH for the converting reaction were 37°C and 6, respectively. Hg^2+^, Zn^2+^, Cu^2+^and SDS inhibited the enzyme activity at different levels whereas Mn^2+ ^showed an enhancing effect by 10%.

**Conclusion:**

In this study, several TreS producing strains were screened from a source of soil bacteria. The characterization of the recombinant TreS of *Enterobacter hormaechei *suggested its potential application. Consequently, a strategy for isolation of TreS producing strains and cloning of novel *treS *genes from natural sources was demonstrated.

## Background

Trehalose, a non-reducing disaccharide with two glucoses linked by a 1, 1-glycosidic linkage, is widespread throughout the biology world. In some lower orders of plants and fungi, this disaccharide is mainly stored as a source for carbon and energy [1]. In the animal kingdom it is abundant and e.g. insects use it as a source for glucose to provide sufficient energy during flight [2]. In yeast and bacteria, it was reported that trehalose could protect cells from a variety of physical and chemical stresses, such as freezing, heat, desiccation, acidic conditions, and osmotic and oxidative stress [3,4]. Due to its desirable characteristics, it has been also applied as an additive, stabilizer and preservative to food, cosmetics, as well as medicinal and biological reagents [5].

At present, many trehalose synthesizing enzymes systems have been reported in microorganisms [6]. There are three pathways widely distributed among them: (1) Phosphate-based enzyme complex systems: Trehalose is synthesized through the transfer of the glucosyl moiety from UDP-glucose to glucose-1-phosphate, forming the intermediate trehalose-6-phosphate which was then hydrolyzed by phosphatase to yield free trehalose [7,8]. (2) A two-step enzyme system with maltooligosyl trehalose synthase (TreY) and maltooligosyl trehalose trehalohydrolase (TreZ) were found for trehalose biosynthesis in *Arthrobacter sp*. [9], *Brevibacterium helvolum *[10] and *Bradyrhizobium *[11]. (3) TreS synthesizing trehalose from maltose in one step was found only in bacteria [12]. We are most interested in TreS as a simple biocatalyst for trehalose production. Up to now, a few *treS *genes from different bacterial species have been reported to be cloned, expressed and characterized, including *Pseudomonas stutzeri *CJ38 [13], *Pimelobacter sp. *R48 [14], *Mycobacterium smegmatis *[15], *Arthrobacter aurescens *[16], *Propionibacterium freudenreichii *[17], *Corynebacterium glutamicum *ATCC13032 [18] and some thermophilic strains [19-21].

The Tibetan plateau is a high-altitude region where it can be assumed that bacteria are frequently exposed to stress conditions, such as cold, hypoxia, nutritional stress and high ultraviolet radiation. The microorganisms in this environment have received much interest because of their special properties [22]. Therefore, it is highly desirable to develop an effective strategy for isolation and subsequent screening of new TreS from the extreme environment

In this study, several TreS producing strains were screened from soil bacteria derived from the plateau soil sample by degenerate PCR based on the analysis of conserved domains. The full-length of the novel *treS *gene of *Enterobacter hormaechei *was obtained by thermal asymmetric interlaced PCR (TAIL-PCR). Subsequently, the gene was expressed in *Escherichia coli *(*E. coli*), and the recombinant TreS was purified and characterized.

## Results and discussion

### Gene sequence analysis and strain screenings

**Figure 1:**
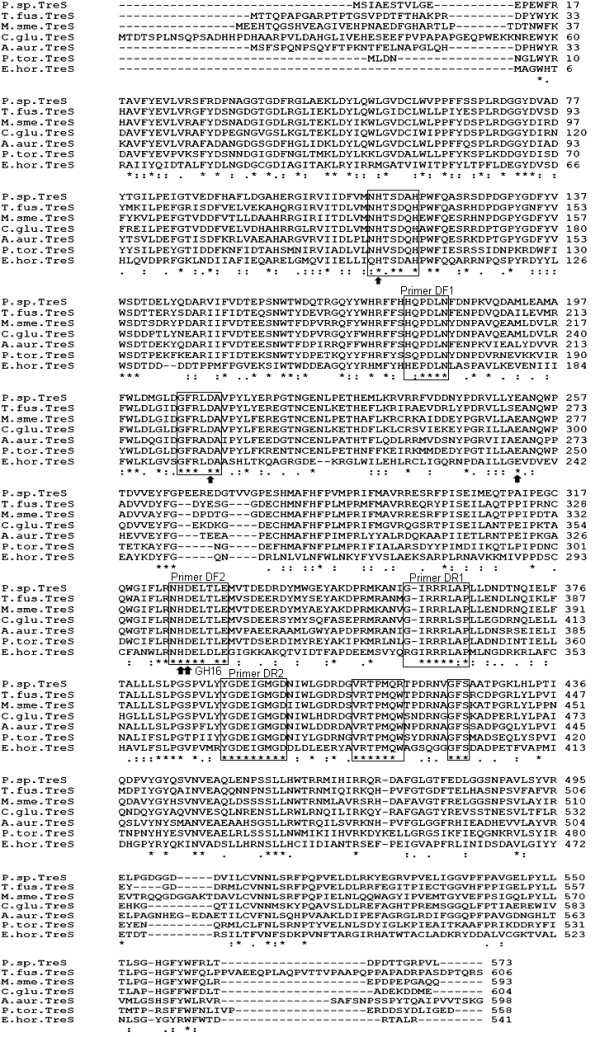
**Multiple sequence alignment of TreS**. P.sp.TreS, *Pimelobacter sp*. R48 TreS (BAA11303); T.fus.TreS, *Thermobifida fusca *TreS (AAZ54622); M.sme.TreS, *Mycobacterium smegmatis *TreS (ABK71531); C.glu.TreS, *Corynebacterium glutamicum *TreS (CAF20645); A.aur.TreS, *Arthrobacter aurescens *TreS (ACL80570); P.tor.TreS, *Picrophius torridus *TreS (AAT42654); E.hor.TreS, *Enterobacter hormaechei *TreS (FJ215664). Conserved residues are in frames. GH16 represents conserved domain of glycoside hydrolase family 16. Arrows indicate putative conserved active sites. Asterisks located under the alignment represent identity; colons represent strong characteristic similarity and periods represent similarity.

By multiple alignment analysis, the four highly conserved motifs HE/QPDLN, NHDELD/TLE, GIRRRLAP and YGDEIGMGD were found among protein sequences of TreS published at NCBI (Figure 1). The NHDELD/TLE domain (the DF2 primer region) had the feature of glycoside hydrolase (GH) family 16 site, suggesting that the identified *treS *gene encoding products possibly had hydrolytic activity. In addition, there were some other motifs identified by sequence alignment with relatively lower homologies, including N/QHTSDQ/AH, GFRL/ADA, VRTPMQW and GGFS (Figure 1).

Among the 86 bacterial strains isolated from the plateau soil sample, 6 different bacterial species were identified to be *treS *positive. Amplified core regions were sequenced and showed different similarity to relevant genes as summarized in Table 1. This result provided evidence for the distribution of TreS producing bacteria in extreme environment.

**Table 1:** Strains identified by 16SrRNA and protein sequence identities

Strain name	Primer set	Amplified size (bp/aa)	Blast result of core regions		
			
			Protein identity	Compared bacterium	Genbank accession No.
*Enterobacter hormaechei*	DF2 & DR2	228/76	69%	*Klebsiella pneumoniae*	CP000964
*Enterobacter asburiae*	DF2 & DR2	228/76	72%	*Klebsiella pneumoniae*	CP000964
*Chryseobacterium sp.*	DF2 & DR2	225/75	96%	*Corynebacterium glutamicum*	YP226546
*Pseudomonas putida*	DF1 & DR1	444/148	98%	*Pseudomonas putida*	ACA72320
*Stenotrophomonas maltophilia*	DF1 & DR2	642/214	99%	*Stenotrophomonas maltophilia*	CAQ46113
*Pseudomonas aeruginosa*	DF1 & DR2	657/219	95%	*Pseudomonas fluorescens*	AAY92145

### Cloning of Enterobacter hormaechei treS gene and analysis

A 228 bp core region of *Enterobacter hormaechei treS *gene was amplified by degenerate PCR. Its 3' and 5' flanking sequences were amplified by TAIL-PCR. By this a 1626 bp open reading frame (ORF) sequence was obtained, encoding 541 amino acids with a predicted molecular mass (M_r_) of 61.8 kDa. As analyzed by NCBI BLAST, the full-length ORF of the *Enterobacter hormaechei treS *gene showed the highest similarity with several encoding sequences of TreS from *Klebsiella pneumoniae *342 (CP000964, 815/1161, 70%), *Pseudomonas stutzeri *A1501 (NC000304, 386/569, 67%) and *Pseudomonas aeruginosa *PA7 (CP000744, 730/1119, 65%).

All of the conserved regions mentioned above were found in *Enterobacter hormaechei *TreS (Figure 1). Besides the GH16 domain, other active sites of glycosidase (H...D...E...HD) were also observed in most bacterial TreS (Figure 1), as reported by Chen [20]. It is also reported that a glycosidase of *Thermobifida fusca *had TreS activity [21]. Therefore, it was suggested that TreS might employ a hydrolysis mechanism.

### Expression and purification of recombinant TreS

**Figure 2:**
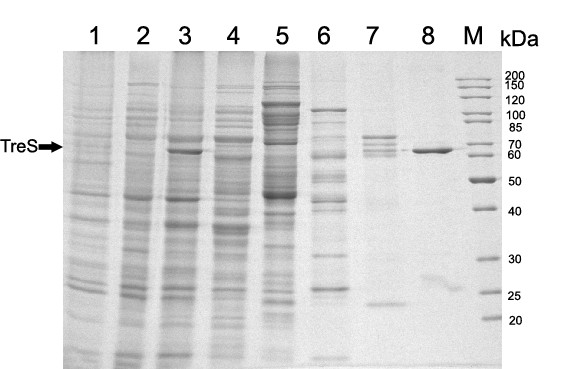
**SDS-PAGE analysis of samples during TreS purification**. All the protein samples were loaded onto a 12.5% polyacrylamide gel under denaturing conditions. The gel was stained with Coomassie Brilliant Blue R-250. The arrow indicates recombinant TreS. Lane 1, control: Cell lysate of *E. coli *BL21 (DE3) plysS with pET30a(+); lane 2, cell lysate before induction; lane 3, cell lysate after induction; lane 4, flow through of the Ni-NTA column; lane 5, wash by 20 mM imidazole; lane 6, wash by 40 mM imidazole; lane 7, wash by 60 mM imidazole; lane 8, purified TreS; lane M, molecular weight marker (Bomaide, Beijing, China).

The pET30a(+)-*treS *plasmid was transformed into the *E. coli *BL21 (DE3) plysS expression host, and cells were induced by isopropyl β-D-1-thiogalactopyranoside (IPTG). When compared to the sample without induction, only the induced cells containing the recombinant vector expressed an extra 65 kDa protein (Figure 2). The recombinant protein was about 3 kDa heavier than the predicted M_r _of 61.8 kDa, which was due to the additional 46 amino acids including the 6-His tag at the N' terminus.

67 mg of recombinant TreS were purified from the supernatant of cell lysate containing 589 mg of crude protein. Soluble recombinant protein was effectively purified at a yield of 11.4% (w/w) of the total soluble protein. The final preparation showed a single band with an M_r _of approximately 65 kDa (Figure 2).

### Activity assay of recombinant TreS and products assay of catalytic reaction

TreS activity was detected with purified TreS in reactions of the conversion between maltose and trehalose. It was confirmed that the cloned fragment was the intrinsically coding sequence of active TreS. The highest activity was calculated to be 18.5 U/mg. The activity value was lower than that of the recombinant TreS of *Pseudomonas stutzeri *CJ38 [13], but close to that of the recombinant TreS of *Mycobacterium smegmatis *[15].

**Figure 3:**
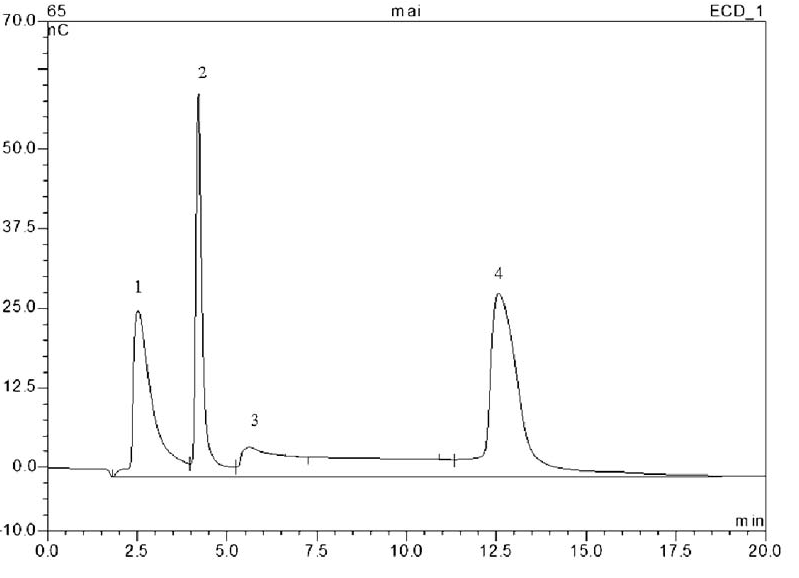
**IC assay of reaction products**. Reaction mixtures containing 400 μl purified TreS solution and 100 μl maltose (500 mM) substrate in 50 mM potassium phosphate buffer (pH 6) were incubated at 37°C for 2 h. The reaction mixtures were subsequently analyzed by IC, as described in the "Methods" section. Peak 1, trehalose; peak 2, glucose; peak 3, buffer reagent; peak 4, maltose.

The amount of glucose was also detected by ion chromatography (IC) (Figure 3) and shown to be consistent with the features of glycoside hydrolase as discussed above. Commonly, glucose is released in most of the conversion reactions of bacterial-derived TreS [14-21]. The small amount of glucose is generated under entry of a water molecule into the catalytic pocket to induce hydrolysis prior to formation of the glycosidic linkage [19]. Individually, TreS from *Pseudomonas stutzeri *CJ38 was the only one reported without generation of glucose as a byproduct [13]. We found relatively low homology between the two TreS from *Enterobacter hormaechei *and *Pseudomonas stutzeri *CJ38 (data not shown), indicating that there exists a structural difference in the functional role. Hence, in order to improve the efficiency of the major trehalose formation, the strategy to screen the enzyme without byproducts still remains to be further exploited.

### The effect of pH and temperature on recombinant TreS activity

**Figure 4:**
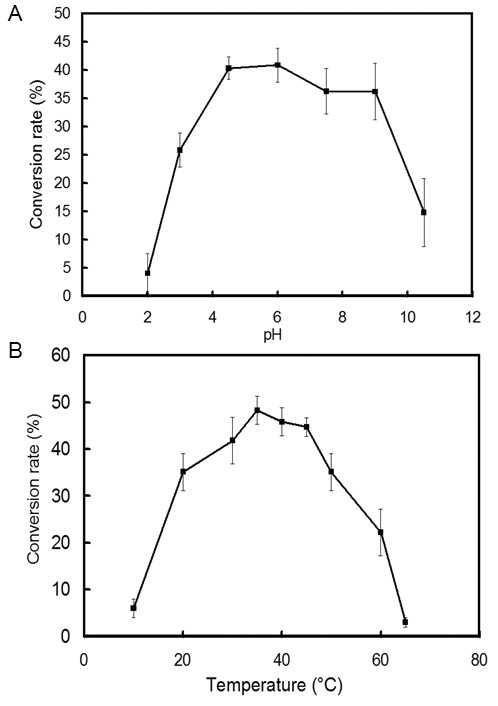
**Effect of pH and temperature on TreS activity**. (A) Effect of pH on TreS activity. The enzyme activity of TreS at various pHs was assayed at 37°C in 50 mM potassium phosphate buffer (pH 2–10.5) for 30 min, using 100 mM maltose as a substrate; (B) Effect of temperature on TreS activity. The enzyme activity of TreS at various temperatures was assayed in 50 mM potassium phosphate buffer (pH 7) for 30 min, using 100 mM maltose as a substrate.

The conversion rate of recombinant TreS was constantly above 30% (equivalent to 65% of the maximal activity) at a broad pH range of 4 to 9, and reached the highest activity at pH 6 (Figure 4A). The maximal conversion rate of 48% was observed at the optimum temperature of 37°C (Figure 4B). The optimum temperature was similar to that of *Pseudomonas stutzeri *CJ38 [13], *Mycobacterium smegmatis *[15], *Arthrobacter aurescens *[16] and *Thermus caldophilus *[23]. Although the conversion from maltose to trehalose could be accelerated at high temperatures (data not shown), TreS could produce more byproduct glucose when the temperature was increased [21,23]. Thus, it is probably more efficient to carry out conversions at moderate temperatures. In this report, the recombinant TreS showed a stable performance under the wide working conditions (pH 4–9 and 20–55°C, Figure 4). It suggested that the enzyme might be a candidate for trehalose production.

### The effects of metal ions and reagents on recombinant TreS activity

Recombinant TreS activity could be influenced by several ions and reagents at different levels. The enzyme was strongly inhibited by Hg^2+^, Zn^2+ ^and Cu^2+ ^for more than 40% and by SDS for about 90%. Other ions and chemical reagent, such as Mg^2+^, Fe^2+^, Na^+^, NH_4 _^+ ^and EDTA, had no obvious effect, whereas Ca^2+ ^and Mn^2+ ^increased the activity slightly (Table 2). Besides, TreS was inactivated by addition of β-mercaptoethanol (β-ME), which supports the hypothesis of a structural dependence on disulfide-bonds.

**Table 2:** Effects of metal ions and chemicals on the activity of recombinant TreS

Reagent	Relative activity (%)	Reagent	Relative activity (%)
None	100*	Cu^2+^	52 ± 4.7
Hg^2+^	57 ± 2.2	Na^+^	90 ± 2.4
Mg^2+^	98 ± 1.3	NH_4 _^+^	101 ± 4.3
Ca^2+^	103 ± 7.9	SDS	10 ± 1.1
Zn^2+^	59 ± 1.3	EDTA	98 ± 5.8
Fe^2+^	92 ± 5.9	β-ME	0
Mn^2+^	111 ± 10.7		

* The enzyme activity of TreS without the addition of ions or reagents was taken to be 100%.

### Kinetics of recombinant TreS activity

The *K*_*m *_values for recombinant *E. hormaechei *TreS were found to be 25 mM for maltose and 75 mM for trehalose. *V*_*max *_values of 1.4 mM/min/mg protein for maltose and 3.2 mM/min/mg protein for trehalose were calculated, respectively. With regard to these results, the recombinant TreS had a higher affinity to maltose and a favorite reaction direction toward the synthesis of trehalose. Interestingly, all reported TreS enzymes share the feature of a reversible conversion at different degrees.

## Conclusion

In summary, some TreS producing strains had been isolated from natural environment based on conserved domains and degenerate PCR. The *treS *gene from *Enterobacter hormaechei *was cloned by TAIL-PCR and expressed successfully. The characterization of the recombinant TreS suggested its potential application for trehalose production from maltose. Thus, the general applicability of this strategy for isolation of TreS producing strains and cloning of novel *treS *genes from natural sources was demonstrated.

## Methods

### Bacterial strains, media and culture conditions

Bacterial strains obtained from a Tibetan Plateau soil specimen were grown and enriched in nutrient broth medium (pepton1% w/v, beef extract 0.3% w/v, NaCl 0.5% w/v) at 30°C for 2 days. *Arthrobacter aurescens*, serving as a TreS positive control, was obtained from China General Microbiological Culture Collection Center (CGMCC1.1892).

*E. coli *Top10 was used for construction of recombinant plasmids. *E. coli *BL21 (DE3) plysS (Novagen, Germany) was used as expression host and grown in LB medium containing 50 μg/ml kanamycin (Kan) and 34 μg/ml chloramphenicol (Cam) after transformation.

### Gene sequence analysis and strain screenings

All bacterial TreS sequences published in the NCBI Database were collected and analyzed by the multiple sequence alignment program CLUSTAL W2 . As shown in Figure 1, four degenerate primers (DF1, 2 and DR1, 2) were synthesized based on the conserved domains (see additional file 1 for the primer sequences).

DNA preparation of bacterial strains isolated from the soil specimen was performed by alkaline lysis [24]. Degenerate PCR was carried out according to the parameters optimized with the genome DNA template of *Arthrobacter aurescens*. PCR products were separated on a 1% agarose gel, recovered by using a Gel Extraction Kit (Tiangen, Beijing, China), and sub-cloned into the pMD18-T vector (TaKaRa, DaLian, China) for sequence analysis (SunBio, Beijing, China). The sequenced fragments were analyzed by BLAST . All isolated positive strains were subsequently identified by their 16SrRNA sequence.

### Cloning of the treS gene of Enterobacter hormaechei and construction of the expression vector

TAIL-PCR was performed to amplify the 3' and 5' fragments of *Enterobacter hormaechei treS*. The PU and PD primers (see additional file 1) for TAIL-PCR were designed and synthesized according to the core region amplified in degenerate PCR. The reaction parameters and the AD primers for TAIL-PCR were referred to previous reports [25,26]. The full length of *treS *was assembled from 3', 5' fragments and the core region. The deduced amino acid sequence was analyzed by DNAMAN 1.0.

The TreSF and TreSR primers (see additional file 1) were synthesized to introduce *Nco *I and *Not *I sites into the 3' and 5' ends of *treS *ORF, respectively. The PCR product was digested by *Nco *I and *Not *I, and inserted into the same digested pET30a(+) vector (Novagen, Cat No. 69909-3, Darmstadt, Germany) to generate His-tagged pET30a(+)-*treS*. The recombinant plasmid was confirmed by DNA sequencing and transformed to the *E. coli *BL21 (DE3) plysS.

### Protein expression and purification

The *E. coli *BL21 (DE3) plysS transformed with pET30a(+)-*treS *was cultured in LB medium containing 50 μg/ml Kan and 34 μg/ml Cam in a shaker at 220 rpm and 37°C until an OD_600 _of 0.6 was reached. These cells were induced with a final concentration of 0.5 mM IPTG and grown at 25°C for an addition of 4 h. Cell extracts were analyzed by 12.5% (w/w) SDS-polyacrylamide gel electrophoresis (SDS-PAGE). Gels with expressed protein were analyzed by a Molecular Imager^® ^Gel DocTM XR system 170–8170 (Bio-Rad, Hercules CA, USA) using the Quantity one-4.6.3 1-D analysis software.

For purification, cells were harvested by centrifugation and resuspended in lysis buffer (50 mM KH_2_PO_4_-K_2_HPO_4_, 500 mM NaCl, pH 7.9) followed by sonification and centrifugation at 12,000 × g for 20 min at 4°C to remove insoluble cell debris. The 6-His tagged protein in supernatant fraction was purified by using a Ni-NTA affinity chromatography column (NEB, Beijing, China). Protein concentrations were determined by the Bradford method using bovine serum albumin as a standard [27].

### Activity assay of recombinant TreS

The catalytic reaction was performed in a mixture containing the TreS solution and 100 mM maltose in 50 mM phosphate buffer (pH 6) at 37°C for 2 h. Samples were taken at different time intervals during the reaction and boiled for 10 min to stop the reaction. The reaction mixtures were separated by thin layer chromatography on G-60 TLC silica plates (Merck, Darmstadt, Germany) using a solvent system of 1-butanol/pyridine/water (4:5:1, v/v). Products released by TreS were quantified by IC using a Dionex2500 system equipped with a CarboPacPA™ 20 column. One unit (U) of TreS was defined as the amount of enzyme required to produce 1 μmol trehalose per min under the specified conditions [23]. The conversion rate was calculated by the ratio of the trehalose product to the amount of maltose substrate.

### Properties of recombinant TreS

The optimum pH of TreS was assayed by incubating the purified enzyme with 100 mM maltose substrate in 50 mM potassium phosphate buffer at pH 2 to 10.5 and 37°C for 30 min, respectively. The optimum temperature for TreS activity was determined at 10 to 60°C using the same buffer at pH 7 for 30 min, respectively. To determine the effect of metal ions and different chemical reagents on TreS, its activity was also assayed in the presence of these ions or compounds at 1 mM, respectively.

### Determination of kinetic parameters

The Michaelis-Menten (*K*_*m*_) and maximum activity (*V*_*max*_) constant for recombinant TreS were determined with different concentrations of substrates ranging from 1 to 200 mM, and the data were plotted according to the Lineweaver and Burk method.

### Nucleotide sequence accession number

The nucleotide sequence of *Enterobacter hormaechei treS *has been submitted to GenBank under accession no. FJ215664.

## Competing interests

The authors declare that they have no competing interests.

## Authors' contributions

MY carried out gene cloning, expression, and characterization of the enzyme, and composed the manuscript. XLW carried out the isolation of strains from soil sample and the degenerate PCR optimization together with MY. WNG performed the TAIL-PCR with genomic DNA of isolated strains. HBD conceived and supervised the study. All authors have read and approved the final manuscript.

## Supplementary Material

Additional file 1 **Primers used in this study**. The nucleotide sequences of the primers used in this study. Click here for file
