# Supplementary material for: Molecular cloning and expression of a novel trehalose synthase gene from Enterobacter hormaechei
Source: Microb Cell Fact. 2009 Jun 12;8:34. doi: 10.1186/1475-2859-8-34 (PMC2701924; doi:10.1186/1475-2859-8-34)
Supplement: Additional file 1 — Primers used in this study. The nucleotide sequences of the primers used in this study. [file 1475-2859-8-34-S1.doc]

**Additional file 1:**

Primers used in this study

| Primer name | Sequence | Size(bp) |
| --- | --- | --- |
| DF1 | 5’-CAYCARCCIGAYCTSAAYTW-3’ | 20 |
| DF2 | 5’-AAYCAYGAYGARYTSACSCTIGA-3’ | 23 |
| DR1 | 5’-AGSGTSARYTCRTCRTGRTT-3’ | 20 |
| DR2 | 5’-TCSCCCATVCCRATYTCRTC-3’ | 20 |
| PU1 | 5’-GCGAAAACAAGACCGCATGGCAAAAG-3’ | 26 |
| PU2 | 5’- CGGATACCTCGCTGATACACACTCAT-3’ | 26 |
| PU3 | 5’-CTGCTTCGCTTTTTTTCCTATCCCT-3’ | 25 |
| PD1 | 5’-GGGATAGGAAAAAAAGCGAAGCAGAC-3’ | 26 |
| PD2 | 5’-GAGTGTGTATCAGCGAGGTATCCG-3’ | 24 |
| PD3 | 5’-GCGTACCGGTCATGCGCTAC-3’ | 20 |
| AD2 | 5’-NGTCGASWGANAWGAA-3’ | 16 |
| AD13 | 5’-WCAGNTGWTNGTNCTG-3’ | 16 |
| TreSF | 5’-TGCCATGGCTGCAGGCTGGCATACACGC-3’ | 28 |
| TreSR | 5’-ATGCGGCCGCTCAGCGCAGTGCGGTACGAT-3’ | 30 |
| T7 promoter | 5’-TAATACGACTCACTATAGGG-3’ | 20 |
| T7 terminator | 5’-GCTAGTTATTGCTCAGCGG-3’ | 19 |
